# Documentation of Screening for Firearm Access by Healthcare Providers in the Veterans Healthcare System: A Retrospective Study

**DOI:** 10.5811/westjem.2021.4.51203

**Published:** 2021-05-19

**Authors:** Cynthia A. Brandt, T. Elizabeth Workman, Melissa M. Farmer, Kathleen M. Akgün, Erica A. Abel, Melissa Skanderson, Bevanne Bean-Mayberry, Qing Zeng-Treitler, Maryann Mason, Lori A. Bastian, Joseph L. Goulet, Lori A. Post

**Affiliations:** * Yale School of Medicine, Department of Emergency Medicine, New Haven, Connecticut; † VA Connecticut Healthcare System, West Haven, Connecticut; ‡ The George Washington University, Biomedical Informatics Center, Washington, District of Columbia; § Center for the Study of Healthcare Innovation, Implementation & Policy (CSHIIP), VA Greater Los Angeles Healthcare System, Los Angeles, California; ¶ Yale School of Medicine, Department of Psychiatry, New Haven, Connecticut; || UCLA David Geffen School of Medicine, Department of Medicine, Los Angeles, California; # Northwestern University, Department of Emergency Medicine, Chicago, Illinois; ** Yale School of Medicine, Department of Internal Medicine, New Haven, Connecticut; †† Northwestern University, Department of Geriatric Medicine, Chicago, Illinois; §§ VA Medical Center, Washington, District of Columbia

## Abstract

**Introduction:**

Presence of a firearm is associated with increased risk of violence and suicide. United States military veterans are at disproportionate risk of suicide. Routine healthcare provider screening of firearm access may prompt counseling on safe storage and handling of firearms. The objective of this study was to determine the frequency with which Veterans Health Administration (VHA) healthcare providers document firearm access in electronic health record (EHR) clinical notes, and whether this varied by patient characteristics.

**Methods:**

The study sample is a post-9–11 cohort of veterans in their first year of VHA care, with at least one outpatient care visit between 2012–2017 (N = 762,953). Demographic data, veteran military service characteristics, and clinical comorbidities were obtained from VHA EHR. We extracted clinical notes for outpatient visits to primary, urgent, or emergency clinics (total 105,316,004). Natural language processing and machine learning (ML) approaches were used to identify documentation of firearm access. A taxonomy of firearm terms was identified and manually annotated with text anchored by these terms, and then trained the ML algorithm. The random-forest algorithm achieved 81.9% accuracy in identifying documentation of firearm access.

**Results:**

The proportion of patients with EHR-documented access to one or more firearms during their first year of care in the VHA was relatively low and varied by patient characteristics. Men had significantly higher documentation of firearms than women (9.8% vs 7.1%; P < .001) and veterans >50 years old had the lowest (6.5%). Among veterans with any firearm term present, only 24.4% were classified as positive for access to a firearm (24.7% of men and 20.9% of women).

**Conclusion:**

Natural language processing can identify documentation of access to firearms in clinical notes with acceptable accuracy, but there is a need for investigation into facilitators and barriers for providers and veterans to improve a systemwide process of firearm access screening. Screening, regardless of race/ethnicity, gender, and age, provides additional opportunities to protect veterans from self-harm and violence.

## INTRODUCTION

In 2020 42% of United States (US) households reported owning a firearm.^1^ Firearms in the home increase risk of violent events,^2^–^5^ and is a significant threat to public health. Nearly half (44.9%) of all US military veterans own a firearm, with ownership reportedly higher among males (47.2%).^6^ Veterans are at disproportionate risk for suicide,^7^ accounting for 20% of suicide deaths despite constituting 13% of the US population. Firearms are involved in 67% of suicides among veterans compared with 50% of the general public.^8^

While access to firearms is associated with increased risk for injury and death, safe firearm storage is associated with decreased risk.^9^,^10^ Public health advocates recommend strategies to restrict access to lethal means as a suicide prevention strategy.^11^ For firearms these processes include safe storage measures such as gun safes, gun locks, storage of ammunition and guns separately, and storage of guns unloaded and locked. 12 Members of the military tend to store firearms unsafely with 45.2% reporting they store firearms both loaded and unlocked, and an additional 33% store firearms either loaded or unlocked.^13^,^14^

Screening veterans for firearms ownership and safe storage is needed to prevent unnecessary injuries and deaths. Healthcare providers are in a position to screen and counsel patients on safe firearm storage.^4^ Counseling on health and safety is a well-established healthcare practice; there are guidelines for screening and counseling in many areas of health including healthy eating, physical activity, mental health, and injury prevention.^15^ While firearm-related injuries and deaths are a public health problem, particularly in the US,^16^ a minority of physicians report engaging in firearm counseling.^17^ Despite several groups having recommended both targeted and universal screening for firearm access,^17^–^24^ there are no current national guidelines for screening in primary care, urgent care or emergency care settings even though gun safety is associated with lower risk of injuries and death.^25^,^26^

To understand how current practice may be adapted, more information on the frequency with which healthcare providers document firearm screening is needed. In this study we present results of Veterans Health Administration (VHA) healthcare providers’ documentation of firearm access screening in electronic health record (EHR) notes among VHA patients in outpatient primary care, urgent care, and emergency department (ED) settings.

## METHODS

The study is a cross-sectional examination of the frequency of documentation of screening for veterans’ access to firearms across several healthcare settings using natural language processing (NLP), which refers to automatic computational processing of human language.^27^ The study was approved by the Veterans Administration Connecticut Healthcare System Institutional Review Board.

**Population Health Research Capsule**

What do we already know about this issue?

*United States Veterans are more likely to own a firearm and to be at risk for firearm injuries and death than civilian populations.*

What was the research question?

*Our study aimed to determine how frequently VA healthcare providers document firearm access screening.*

What was the major finding of the study?

*Documentation of firearm access for Veterans by healthcare providers was low but higher in men than women.*

How does this improve population health?

*Identifying barriers and facilitators to help healthcare providers increase screening for firearms and counsel safe storage could support prevention efforts.*

The study sample included men and women veterans from a national, post-9–11 cohort^28^,^29^ during their first year of VHA healthcare, defined by the presence of at least one primary care visit from 2012–2017. We obtained data on demographic and veteran military service characteristics from the Defense Manpower Data Center–Contingency Tracking System Deployment File, provided to the VHA from the US Department of Defense. Variables included age, gender, race/ethnicity, marital status, education, rank (e.g., officer, enlisted), military branch (e.g., Army, Marine Corps), and deployment dates. VHA visit information came from EHR data extracted from the Corporate Data Warehouse (CDW). The CDW includes information on healthcare utilization, pharmacy, laboratory, vital signs, coded diagnostic and procedural data (*International Classification of Diseases*, 9th and 10th revisions, Clinical Modification [ICD-9-CM and ICD-10-CM]) and Current Procedural Terminology (associated with all VHA inpatient and outpatient encounters.^30^,^31^

We identified comorbid conditions using ICD-9 and ICD-10 coded diagnoses defined by ≥2 outpatient (on separate days) or ≥1 inpatient code for the condition. This methodology has been used for the identification of psychiatric disorders in administrative data^32^ and human immunodeficiency virus in Medicaid data.^33^ Diagnostic code groupings were previously validated.^34^ Major mental health diagnoses included post-traumatic stress disorders (PTSD), major depressive disorders, alcohol use disorders, and substance use disorders.

### Natural Langauge Processing Tool Development and Performance for Firearm Access Identification

#### Firearm Taxonomy

For the information extraction process, we developed a coding manual for chart review and a taxonomy for firearms for annotation. A taxonomy was created by by searching existing vocabularies (NCBIO, UMLS, SnoMed) and the literature for published ontologies used for guns, gunlock, and firearms. A Cochrane review on gunshot wounds contained terms such as trauma* or injur* or penetrat* or wound*or perforat* or stab* or gunshot or shot, and the Medical Subject Headings (MeSH) database included the following: “Wounds, Gunshot”[Mesh]) OR “Wounds, Penetrating”[Mesh:NoExp]) OR“Multiple Trauma”[Mesh])) OR “polytrauma.” This list of terms was supplemented with over 120 candidate terms and phrases contained in a national database^35^ on firearm homicides. We then reviewed and narrowed the phrases down to 27 (shown in Table 1) deemed relevant by VHA clinicians.

#### Annotation

We identified 2,584,607 notes with one or more of the phrases, and annotated 1856 text snippets randomly selected from notes that contained any of the search terms. Each snippet contains a 35-word span before and after a firearm-relevant phrase. The annotation classifications for firearm access were the following: positive (ability to determine that the veteran had current access to at least one firearm); negative (language that the veteran did not have current access to any firearms); and ambiguous (there was insufficient evidence for either a positive or negative classification from the note – an example might be that the veteran owned a firearm but it was somewhere else). Each snippet was annotated by two of the authors and disagreement adjudicated by their consensus. An inter-annotator agreement was calculated. The annotated snippets served as the reference standard in training and testing.

#### Features

We used n-grams as features. In clinical text, unigrams are single words, and bigrams are two words that occur in a sequence. For example, in the phrase “patient owns a shotgun” the unique unigrams are *patient*, *owns*, *a*, and *shotgun*. In the same phrase, *patient_owns*, *owns_a*, *a_shotgun* are unique bigrams. Alpha or numeric tokens (discrete words and numbers) were counted in the unigrams and bigrams. The features included unique unigrams with a frequency greater than 34, and unique bigrams in the annotation spans with a frequency greater than four. These threshholds are empirically chosen to filter out the less prevalent n-grams and reduce overfitting. The training features for the model (for each document) consisted of binary indications of the presence of each of the identified unigrams and bigrams, along with the offset location of the keyphrase in the snippet.

#### Training and Testing

We used the annotated snippets to train a random forest model with 200 estimators or trees. The random forest model maximum depth was set to 15, with maximum features automatically determined by the model and the gini split criterion. Hyperparameters were determined through gridsearch and other testing. We split the 1856 text snippets into 85% for training and 15% for testing. The model performance was measured by accuracy.

#### Validation

**Figure 1:**
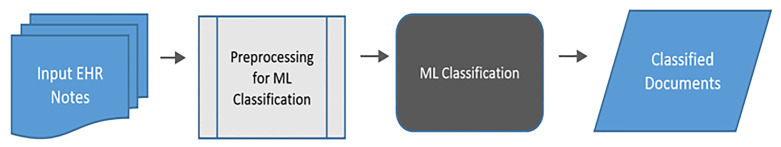
Application pipeline. *EHR*, electronic health record; *ML*, machine learning.

For validation, we annotated an additional 238 clinical notes on the note (instead of the snippet) level, with 175 negtive and 63 positive for firearm access. The random forest model was applied to these notes, based on the snippet identified in each document. Figure 1 below is a schematic of this process.

### Statistical Analysis

We conducted all statistical analyses using SAS software version 9.4 (SAS Institute, Cary, NC). Baseline characteristics of veterans include frequency (percentages) and means (± standard deviations) or median (interquartile range), and differences by age, race, ethnicity and gender were examined using chi-squared test or Student’s t test, as appropriate. We used a multivariable logistic regression model to assess firearm mention and adjust for potential confounding based on the literature. Among those with firearm mention, a logistic model was run to assess firearm access. We examined model fit using quasi-information criterion and residual plots. Hypotheses were tested at a two-sided significance level of α = 0.05.

## RESULTS

The Cohen kappa score measuring inter-annotator agreement among the review team members identifying screening documentation incidents was 80%. On the testing dataset (15%), the accuracy was 81.0%. On the final validation dataset, the random forest model achieved 81.9% accuracy, 90.9% specificity, 57.1% sensitivity, and positive predictive value of 69.2% in classifying the 238 test notes. Table 1 demonstrates the frequency of the most common firearm-related terms within the VHA text notes. These counts are non-distinct by patient but demonstrate the breadth of terms used in clinical notes by providers; many of the highly specific terms were present in notes as historical and exposure events for PTSD documentation, and/or noise exposure (out of 105,316,004 outpatient care notes).

We included data during the first year in VHA care for 762,953 veterans in the analytic sample. Table 2 demonstrates the frequency of documentation of access to firearms and other guns by clinicians within one year of entry into VHA healthcare. The mention of any firearm within a clinical note for veterans was 9.8% of men, 7.1% of women, and 6.5% in veterans over 50 years of age. Among the small number of veterans with any firearm term present, only 24% were classified as positive for access to a firearm (24.7% of men and 20.9% of women [data not otherwise shown]). Prevalence patterns by race of any mention/positive access were similar, with the highest rates among Whites (9.6% mention and 26.3% access [data not otherwise shown]). Documentation of firearms was higher in veterans with higher numbers of mental health visits, emergency and urgent care visits than primary care. Documentation of firearms did not vary regardless of the number of primary care visits (data not shown). After adjustment for demographics, utilization, and comorbidities, significant differences in documentation of access remained by age, gender, and among veterans with major depression or PTSD diagnoses.

## DISCUSSION

Results demonstrate documentation of firearm access in clinical notes for less than 10% of contemporary veterans within the first year of enrollment in VHA healthcare, and that nearly one quarter of those with documentation were identified as having access to a firearm. There was a significantly lower rate of documented access for women veterans, despite data that show high rates of both men and women veterans who live in homes with firearms, and increasing rates of fiream-related suicides among women veterans.^6^,^36^ While documentation does not always equate with conversations between providers and patients, the low frequency of documented patient-provider interactions seen in this population suggests that there is a clear opportunity to increase initiation of conversations about firearm access and safety. Barriers to implementation of firearm screening and safety counseling include provider uncertainty about the effectiveness of firearm screening, provider uncertainty about the legality of asking about firearm ownership, and provider unfamiliarity with firearms. Further, provider unfamiliarity with lethal means restriction as a firearm suicide prevention strategy may prohibit uptake of screening and counseling.^17^,^23^,^37^–^39^ These barriers indicate a need for increased training of healthcare providers on firearm screening and safety counseling and normalizing the opportunities to discuss firearms in a population that has higher rates of firearm ownership and use.

Discussions must be acceptable to providers and to patients for it to be effective. Roszko and colleagues’ review of 53 studies of non-veteran clinician firearm attitudes and practices found that positive attitudes toward firearm discussions were higher than actual documented discussions, with low firearm discussions across all disciplines.^17^ This is encouraging in that it could indicate healthcare providers may be willing to undergo training in initiating and carrying out these discussions, although it remains to be seen whether attitudes differ among VA providers.

While providers may have positive attitudes toward firearm screening and counseling, recent studies show mixed support by gun owners and veterans for healthcare provider initiation of gun safety conversations.^11^,^40^ This suggests that while providers may be willing to initiate these discussions, it is not clear that patients will welcome or participate in them if initiated. Such conversations will need to be clearly delineated as prevention oriented for gun-owning citiziens and families with specific, evidence-based practices such as the following: Homes with locked guns are less likely to have unintentional or self-inflicted injuries with firearms or deaths.^41^

Perhaps related to the reasons specified above, the evidence for the effectiveness of this firearm safety conversation in the clinical setting is mixed.^42^,^43^ For this reason, appropriate, acceptable communication must be used and evaluated to maximize the impact and inform the knowledge base of these efforts in the clinical setting.^44^,^45^ However, a recent epidemiologic review indicates that counseling combined with safety-device provision can impact safe storage in the community.^41^ Promising strategies include following the guiding principles of shared decision-making, with providers stating neutral risks and protective factors related to gun safety and involving gun owners in the development of messaging.^46^,^47^ These neutral risks can be culled from the US Centers for Disease Control and Prevention data as simple facts for patients to understand and acknowledge as part of their gun ownership responsibilities.^48^

Specific to the VHA, an appropriate clinical response to the public health problem of firearm suicide in the veteran population is needed. Further research within the VHA is needed to determine the healthcare setting(s) and provider types most appropriate for firearm screening and counseling interventions. This step will require a participatory approach among health services and informatics researchers to improve the feasibility, acceptability, relevance, and sustainability of interventions.^49^–^52^ In addition, research is needed to determine the modality and intervention format (electronic, face to face, written) that are most effective for each of the key domains in firearm injury research. Data on the moderators of acceptability and effectiveness (demographics, political views, comorbidities, etc.) of screening and interventions from the veteran and provider perspectives are needed. Only then can researchers begin to measure the short- and longer-term outcomes of such interventions and policies. While this approach is clearly specific to the clinical context and persons involved for veteran prevention with firearms, prevention is likely best on the frontlines of care and where repeated encounters occur with trust-building relationships. Thus, primary care, mental health and ED settings/providers may need to partner with the health services and health informatics researchers to fully address the scope of this need and develop interventions that fit the veteran patients and the VHA system. Equipped with information and curiosity, clinicians can engage their veteran patients as part of routine care, instead of urgent or emergent care, and the health services and health informatics teams can inform us about which methods are most feasible and impactful for veteran quality of life and provider use and sustainability.

## LIMITATIONS

Given retrospective studies may introduce sampling bias^53^, we included the entire population, not a sample. The results of the NLP algorithm were limited for the first year of entry into VHA healthcare for years 2012–2017, which might underestimate firearm documentation. The identification of firearm documentation for patterns such as temporal changes, variations in types of providers and provider settings, and other patient characteristics will be explored in future work. For example, in this sample there were increases by year (from 3% in 2012 to 21% in 2017). Further research is needed to help explain this increase.

## CONCLUSION

Natural language processing methods are able to determine the prevalence of documented firearm screening and safety counseling across a large population of US military veterans. We identified low prevalence of firearm access screening documentation and believe that further investigation into facilitators and barriers is necessary. This work should inform the process for development of systemwide practices to reduce firearm suicide and injury among US veterans, a large group at elevated risk.

## Figures and Tables

**Table 1:** Counts of firearm-related terms found in notes (N = 27 terms).

Term	Count	Term	Count
Rifle	45,897	38 caliber	58
Pistol	32,893	9 mm Beretta	49
Shotgun	25,761	Arms dealer	51
12 gauge	848	Blue suicide	3
9 mm Glock	59	Home invasion	2,940
45 caliber	417	Minigun	6
22 caliber	281	Mossberg shotgun	6
Semiautomatic	90	Pistol whip	105
357 Magnum	140	Revolver	2,295
M1 rifle	4	Ruger pistol	5
Gun	653,308	Smith and Wesson	253
Guns	423,119	Sniper rifle	370
Firearm	305,766	Winchester rifle	8
Firearms	1,089,875		

*mm*, millimeter.

**Table 2:** Documentation of access to firearm by covariates with adjusted models for any firearm documentation and access.

	Any documentation, n= 762,953			Adjusted model 1		Adjusted model 2	

	No	Yes		Mention		Access among any documentation	

	N = 690,599 (91%)	No access, N = 54,672 (76%)	Access, N = 17,601 (24%)	OR (95% CI)	P-value	OR (95% CI)	P-value
Gender							
Female	90,282 (13.07)	5,451 (9.97)	1,440 (8.18)	1	n/a	1	n/a
Male	600,398 (86.93)	49,221 (90.03)	16,161 (91.82)	1.45 (1.41, 1.50)	<0.001	1.19 (1.12, 1.27)	<0.001
Age groups, n (%)							
<30	80,598 (11.67)	6,116 (11.19)	1,927 (10.95)	1	n/a	1	n/a
30–49	471,218 (68.23)	41,083 (75.15)	13,515 (76.8)	0.88 (0.86, 0.90)	<0.001	0.99 (0.94, 1.05)	0.008
50+	9,623 (20.1)	7,467 (13.66)	2,156 (12.25)	0.61 (0.59, 0.63)	<0.001	0.89 (0.82, 0.95)	<0.001
Race/ethnicity, n (%)							
White	438,847 (63.54)	34,402 (62.92)	12,247 (69.58)	1	n/a	1	n/a
Black	123,115 (17.83)	10,246 (18.74)	2,607 (14.81)	1.04 (1.02, 1.06)	0.0002	0.73 (0.69, 0.76)	<0.001
Hispanic	80,443 (11.65)	6,651 (12.17)	1,738 (9.87)	0.96 (0.93, 0.98)	<0.001	0.72 (0.68, 0.76)	<0.001
Other	48,275 (6.99)	3,373 (6.17)	1,009 (5.73)	1.02 (0.99, 1.06)	0.2	0.84 (0.78, 0.91)	0.2
MDD, n (%)	147,787 (21.4)	21,949 (40.15)	8,154 (46.33)	1.43 (1.40, 1.45)	<0.001	1.25 (1.20, 1.30)	<0.001
PTSD, n (%)	277,536 (40.18)	38,082 (69.66)	13,300 (75.56)	2.24 (2.20, 2.29)	<0.001	1.25 (1.20, 1.30)	<0.001
Smoking, n (%)							
Never	266,593 (41.14)	18,245 (34.01)	5,438 (31.23)	1	n/a	1	n/a
Past	286,280 (44.18)	28,707 (53.51)	9,631 (55.32)	1.08 (1.06, 1.10)	<0.001	1.05 (1.00, 1.09)	0.7
Current	95,102 (14.68)	6,695 (12.48)	2,341 (13.45)	0.99 (0.96, 1.01)	<0.001	1.11 (1.05, 1.17)	0.002
Chronic pain, n (%)	62,808 (9.09)	8,755 (16.01)	2,991 (16.99)	1.06 (1.03, 1.09)	<0.001	1.03 (0.98, 1.08)	0.3
TBI screen, n (%)	616,836 (89.31)	51,867 (94.87)	16,874 (95.87)	1.04 (0.99, 1.09)	0.09	0.99 (0.89, 1.09)	0.8
MST screen, n (%)	630,124 (91.23)	52,330 (95.72)	16,971 (96.42)	0.91 (0.87, 0.96)	0.0007	0.92 (0.83, 1.03)	0.1
Bipolar, n (%)	24,226 (3.51)	4,394 (8.04)	1,414 (8.03)	1.09 (1.05, 1.12)	<0.001	0.95 (0.89, 1.01)	0.1
OUD, n (%)	215,791 (31.24)	27,274 (49.89)	9,269 (52.66)	1.08 (1.06, 1.10)	<0.001	1.01 (0.97, 1.05)	0.6
Alcohol, n (%)	62,690 (9.08)	11,203 (20.49)	4,049 (23.00)	1.18 (1.16, 1.21)	<0.001	1.1 (1.05, 1.15)	<0.001
Drug, n (%)	63,683 (9.22)	11,743 (21.48)	3,696 (21.00)	0.96 (0.94, 0.99)	0.003	0.80 (0.76, 0.84)	<0.001
# ED visits, mean (SD)	0.30 (0.91)	0.59 (1.46)	0.60 (1.38)	1.03 (1.03, 1.04)	<0.001	0.99 (.098, 1.00)	0.07
# MH visits, mean (SD)	3.32 (8.93)	11.53 (17.70)	12.66 (16.63)	1.03 (1.03, 1.03)	<0.001	1.00 (1.00, 1.00)	0.0002

In descriptive statistics, all variables were significant at p<0.05, except Bipolar and Drug. Models were adjusted for # of ER and MH visit at baseline; 43,921(5%) were missing smoking.

*OUD*, opioid use disorder; *OR*, odds ratio; *CI*, confidence interval; *MDD*, major depressive disorder; *PTSD*, post-traumatic stress disorder; *ED*, emergency department; *MH*, mental health; *SD*, standard deviation; *TBI*, traumatic brain injury; *MST*, military sexual trauma.
